# Prevalence and risk factors of Severe Acute Respiratory Syndrome Coronavirus 2 infection in women and children in peri-urban communities in Pakistan: A prospective cohort study

**DOI:** 10.7189/jogh.12.95955

**Published:** 2022-12-17

**Authors:** Nadia Ansari, Muhammad I Nisar, Farah Khalid, Usma Mehmood, Asra A Usmani, Fariha Shaheen, Aneeta Hotwani, Kehkashan Begum, Amina Barkat, Sachiyo Yoshida, Alexander A Manu, Sunil Sazawal, Abdullah H Baqui, Rajiv Bahl, Fyezah Jehan

**Affiliations:** 1 Department of Pediatrics and Child Health, The Aga Khan University, Karachi; 2 Department for Maternal, Child, Adolescents and Ageing Health, World Health Organization, Geneva, Switzerland; 3 Department of Epidemiology and Disease Control, University of Ghana School of Public Health, Legon, Accra, Ghana; 4 Center for Public Health Kinetics, Global Division, LGL Vinoba Puri, Lajpat Nagar II, New Delhi, India; 5 Public Health Laboratory-IDC, Chake Chake, Pemba, Tanzania; 6 Department of International Health, Johns Hopkins Bloomberg School of Public Health, Baltimore, Maryland, USA

## Abstract

**Background:**

Population-based seroepidemiological surveys provide accurate estimates of disease burden. We compare the COVID-19 prevalence estimates from two serial serological surveys and the associated risk factors among women and children in a peri-urban area of Karachi, Pakistan.

**Methods:**

The AMANHI-COVID-19 study enrolled women and children between November 2020 and March 2021. Blood samples were collected from March to June 2021 (baseline) and September to December 2021 (follow-up) to test for anti-SARS-CoV-2 antibodies using ROCHE Elecsys®. Participants were visited or called weekly during the study for recording symptoms of COVID-19. We report the proportion of participants with anti-SARS-CoV-2 antibodies and symptoms in each survey and describe infection risk factors using step-wise binomial regression analysis.

**Results:**

The adjusted seroprevalence among women was 45.3% (95% confidence interval (CI) = 42.6-47.9) and 82.3% (95% CI = 79.9-84.4) at baseline and follow-up survey, respectively. Among children, it was 18.4% (95% CI = 16.1-20.7) and 57.4% (95% CI = 54.3-60.3) at baseline and follow-up, respectively. Of the women who were previously seronegative, 404 (74.4%) tested positive at the follow-up survey, as did 365 (50.4%) previously seronegative children. There was a high proportion of asymptomatic infection. At baseline, being poorest and lacking access to safe drinking water lowered the risk of infection for both women (risk ratio (RR) = 0.8, 95% CI = 0.7-0.9 and RR = 1.2, 95% CI = 1.1-1.4, respectively) and children (RR = 0.7, 95% CI = 0.5-1.0 and RR = 1.4, 95% CI = 1.0-1.8, respectively). At the follow-up survey, the risk of infection was lower for underweight women and children (RR = 0.4, 95% CI = 0.3-0.7 and RR = 0.7, 95% CI = 0.5-0.8, respectively) and for women in the 30-39 years age group and children who were 24-36 months of age (RR = 0.6, 95% CI = 0.4-0.9 and RR = 0.7, 95% CI = 0.5-0.9, respectively). In both surveys, paternal employment was an important predictor of seropositivity among children (RR = 0.7, 95% CI = 0.6-0.9 and RR = 0.8, 95% CI = 0.7-1.0, respectively).

**Conclusion:**

There was a high rate of seroconversion among women and children. Infection was generally mild. Parental education plays an important role in protection of children from COVID-19.

Since the start of the COVID-19 pandemic, there have been more than 462 million confirmed COVID-19 cases and over 6 million deaths [1]. Pakistan reported its first COVID-19 case on February 26, 2020. Thereafter, more than 1.5 million cases and around 30 000 deaths have been reported [2]. Case detection relies traditionally on clinical surveillance whereby an individual after developing signs and symptoms of a disease seeks care, is examined by a clinician who orders the appropriate test and is diagnosed as having the disease based on the test result [3,4]. Since a large proportion of SARS-CoV-2 infections are mild or asymptomatic, relying solely on the clinical surveillance will lead to underestimation of the true extent of infection in the population [5]. Population-based serosurveys, which look for evidence of past infection by detecting antibodies against a disease, provide an alternative approach to estimate the true burden of infection [6].

The Aga Khan University (AKU), with funding from Bill & Melinda Gates Foundation, and in collaboration with World Health Organization (WHO), Geneva, established a cohort of 2500 pregnant women from low socio-economic peri-urban communities in Karachi, Pakistan. This cohort, known as the Alliance for Maternal and Newborn Health Improvement (AMANHI) is part of a multi-country initiative launched between 2014-2018 in Bangladesh, Tanzania, and Pakistan. Pregnant women were enrolled between 13-≤20 weeks of gestation, biological samples and phenotypical information were collected at different time points and outcome of the pregnancy was recorded. Mother and newborns were then followed for growth, development, and mortality outcomes. Detailed methodology and characteristics of the cohort have been described previously [7,8].

This well-characterized cohort provided us an opportunity to quickly conduct a seroprevalence survey immediately before the COVID-19 vaccine rollout to these age groups in Pakistan and again, at the end of December 2021. The primary aim of this study was to estimate the age-specific prevalence of COVID-19, through antibody assays, and its risk factors among women of reproductive age group and their young children who were part of the AMANHI cohort in Pakistan.

## METHODS

### Study participants and sample collection

This was a cross-sectional survey carried out in Ibrahim Hyderi, a peri-urban area in Karachi, Pakistan. The study area is a densely populated coastal community with a total population of 78 029 where fishing is the main source of livelihood. All women and their children enrolled in the AMANHI biobank study who were residing in the study area at the time of the study were eligible to be part of this survey [8]. Participants were approached between March and June 2021 to take part in the first serosurvey (baseline survey). A second round of survey was conducted from September to December 2021 (follow-up survey).

After obtaining written informed consent, a baseline questionnaire was administered for the mother and child that covered socio-economic information, history of prior exposure or infection, use of non-pharmacological interventions (NPI’s), nutritional status and co-morbidities. A trained phlebotomist in personal protective equipment (PPE), using all safety precautions and aseptic techniques, collected a 3-5 ml venous blood sample in EDTA tubes from mothers and their children. Women who reported fever and cough or any three of the following symptoms viz fever, cough, sore throat, fatigue, headache, runny nose, loss of taste or smell, nausea, vomiting, and diarrhoea, were referred to the nearest COVID-19 diagnostic testing facility [9].

ROCHE Elecsys® Anti-SARS-CoV-2 assay kit (ROCHE, Basel, Switzerland) and ROCHE Cobas e411 automated analyser were used to detect antibodies against SARS-CoV-2 in the patient’s serum. The assay qualitatively detects total polyclonal antibodies (including IgG) against SARS-CoV-2 nucleocapsid (N) antigen and has a sensitivity and specificity of 88.5% and 100% respectively after 14 days of infection [10,11]. Before each batch, the assay was calibrated using positive and negative quality controls. Assay values above the cutoff of 1.0 were regarded as reactive for the presence of anti-SARS-CoV-2 antibodies.

### Statistical analysis

Descriptive statistics are presented as mean ± standard deviation (SD) for continuous variables and frequencies with percentages for categorical variables. Seroprevalence was estimated using Bayesian estimation method adjusted for test kit accuracy. This approach accounts for uncertainty due to finite laboratory validation data, and produces estimates using typical choices of uninformative or weakly informative prior distributions [12-15]. For univariate and multivariate analyses, binomial logistic regression was used to examine the association between seropositivity and socio-economic factors, nutritional factors, and preventive measures. Stepwise backward elimination was used to retain the covariates significant at a *P*-value ≤0.20 were included in a multivariate model. Variables that remained significant at a *P*-value ≤0.05 that were retained in the final adjusted model. All analysis were performed using Stata version 17.0 (STATA Cooperation, Texas, USA) [16].

## RESULTS

Of the 2500 women and 2352 newborns enrolled in the original AMANHI biobanking cohort, 1960 women and 1771 children were still residing in the catchment area when this study was initiated. All were approached, of which 1414 (72.1%) women and 1097 (62.0%) children took part in the baseline survey and 1142 (58.3%) and 1063 (60.0%) in the follow-up serosurvey. One sixty-eight women were pregnant at the time of enrolment for baseline survey, of whom, 158 had delivered by the follow-up survey, with 139 new pregnancies identified during the study follow-up period.

The mean age of women was reported to be 29.33 ± 5.51, and most of them (55.7%) were aged between 30-39 years. Half of the women were not educated 735 (52.0%) and were housewives. A comparison of characteristics of women who took part in the serosurvey vs who did not is given in Table S1 in the **Online Supplementary Document**.

All collected samples were adequate for biochemical analysis. The overall adjusted seroprevalence among women in baseline survey was 45.3% (95% confidence interval (CI) = 42.6-47.9). Among non-pregnant women, the seroprevalence was 46.0% (95% CI = 43.1-48.9), while it was 39.8% (95% CI = 32.6-47.3) among pregnant women (**Table 1**). Out of 1097 sample collected from children in baseline survey, 203 were seropositive, giving an adjusted seroprevalence of 18.4% (95% CI = 16.1-20.7). Seroprevalence among girls was slightly higher as compared to boys, 19.8% (95% CI = 16.5-23.1) and 17.1% (95% CI = 13.9-20.2), respectively. No significant difference was seen across age groups (**Table 2**).

**Table 1:** SARS-CoV-2 seroprevalence in women of reproductive age enrolled in the Alliance for Maternal and Newborn Health Improvement (AMANHI)-COVID-19 study cohort (n = 1414)

	Baseline survey			Follow-up survey		
	**Total women**	**Non-pregnant women**	**Pregnant women**	**Total women**	**Non-pregnant women**	**Pregnant women**
**Serum samples processed**	**n = 1414**	**n = 1246**	**n = 168**	**n = 1142**	**n = 1003**	**n = 139**
Adjusted seroprevalence, % (95% CIs) *	45.3 (42.6-47.9)	46.0 (43.1-48.9)	39.8 (32.6-47.3)	82.3 (79.9-84.4)	82.7 (80.3-85.0)	78.0 (70.4-84.3)
Seroprevalence, % (95% CIs) by age (in years)*						
20-29	**n = 505**	**n = 426**	**n = 79**	**n = 408**	**n = 346**	**n = 62**
	46.1 (41.5-50.4)	47.6 (42.9-52.4)	38.1 (28.1-48.9)	83.3 (79.3-86.7)	82.9 (78.4-86.6)	84.3 (74.2-91.9)
30-39	**n = 787**	**n = 704**	**n = 83**	**n = 624**	**n = 551**	**n = 73**
	44.3 (40.8-47.7)	44.5 (40.7-48.2)	43.4 (32.8-53.6)	80.4 (77.1-83.4)	81.5 (79.9-93.3)	71.8 (60.8-81.4)
≥40	**n = 122**	**n = 116**	**n = 6**	**n = 110**	**n = 106**	**n = 4**
	48.4 (39.7, -57.1)	49.9 (40.9-58.8)	25.2 (3.4-57.9)	86.5 (79.2-92.1)	87.7 (79.9-93.3)	66.5 (26.8-93.8)

*Adjusted by kit accuracy using Bayesian estimation with sensitivity = 1 and specificity = 0.998 (provided by the kit manufacturer).

**Table 2:** SARS-CoV-2 seroprevalence in children enrolled in the Alliance for Maternal and Newborn Health Improvement (AMANHI)-COVID-19 study cohort (n = 1097)

Seroprevalence in children	Baseline survey	Follow-up survey
Sero-samples processed % (95% CIs)*	**n = 1097**	**n = 1063**
	18.4 (16.1-20.7)	57.4(54.3-60.3)
Child gender % (95% CIs)*		
**Males**	**n = 565**	**n = 545**
	17.1 (13.9-20.2)	57.1 (52.8-61.0)
**Females**	**n = 532**	**n = 518**
	19.8 (16.5-23.1)	57.6 (53.2-61.7)
**Child age, months % (95% CIs) ^ᵻ^ by age**		
**24-36**	**n = 340**	**n = 86**
	17.4 (13.5-21.6)	51.0 (39.9-61.6)
**37-48**	**n = 246**	**n = 398**
	19.6 (14.9-24.7)	54.6 (49.6-59.4)
**≥49**	**n = 511**	**n = 579**
	18.7 (15.5-22.1)	60.1 (55.9-63.9)

*Adjusted by kit accuracy using Bayesian estimation with sensitivity = 1 and specificity = 0.998 (provided by the kit manufacturer).

For the follow-up survey, the overall adjusted seroprevalence among women was 82.3% (95% CI = 79.9-84.4), while it was 82.7% (95% CI = 80.3-85.0) among non-pregnant women and 78.0% (95% CI = 70.4-84.3) among pregnant women (**Table 1**). For children in the follow-up survey, the adjusted overall seroprevalence was 57.4% (95% CI = 54.3-60.3). Seroprevalence increased with age; it was highest among children aged >49 months, 60.1% (95% CI = 55.9-63.9). However, there was no difference between the genders. (**Table 2**).

Paired samples (baseline and follow-up) were available for 981 women to examine seroconversion (**Table 3**), of which 404 (74.4%) women who were previously seronegative tested positive at the follow-up survey, conducted approximately after 3-6 months. From the 438 women who were initially seropositive, 399 (91.1%) remained seropositive at the follow-up. Similarly, in children, of the 877 paired samples, 365 (50.4%) previously seronegative children tested seropositive in the follow-up survey, while 135 (89.4%) of previously seropositive children remained seropositive (**Table 3**). In both surveys, a majority of participants who were positive for anti-SARS-CoV-2 antibodies did not report any symptoms. At the baseline survey, 84.9% of women and 81.8% of children were asymptomatic, and 80.9% of women and 83.3% of children were asymptomatic in the follow-up survey (**Table 4**). At baseline, headache and fever were the most common symptoms among women (Table S2a in the **Online Supplementary Document**) while headache and body ache were more commonly reported symptoms at the follow-up survey (Table S2b in the **Online Supplementary Document**). Among children, runny nose and cough were the most common symptoms at baseline (Table S3a in the **Online Supplementary Document**), while at the follow-up, more children reported fever and cough (Table S3b in the **Online Supplementary Document**).

**Table 3:** Seroconversion from baseline to follow-up survey in women and children

Women	Follow-up survey		
**Baseline survey**	**Non-reactive**	**Reactive**	**Total**
Seronegative	139 (25.6%)	404 (74.4%)	543
Seropositive	39 (8.9%)	399 (91.1%)	438
Total	178	803	981
**Children**	**Follow-up line survey**		
**Baseline survey**	**Non-reactive**	**Reactive**	**Total**
Seronegative	359 (49.7%)	365 (50.4%)	724
Seropositive	18 (11.6%)	135 (89.4%)	153
Total	377	500	877

**Table 4:** Distribution of symptoms by seropositivity among women and children at the baseline and follow-up surveys

	Baseline survey		Follow-up survey	
	**Symptomatic n (%)**	**Asymptomatic n (%)**	**Symptomatic n (%)**	**Asymptomatic n (%)**
**Women**				
Seropositive	94 (14.6%)	545 (84.9%)	206 (18.3%)	909 (80.9%)
Seronegative	153 (19.8%)	615 (79.6%)	47 (18.6%)	205 (81.3%)
**Children**				
Seropositive	30 (17.8%)	166 (81.8%)	100 (16.4%)	503 (82.3%)
Seronegative	137 (15.3%)	745 (83.3%)	68 (15.0%)	379 (83.8%)

In the final adjusted multivariate regression model, in baseline survey, being in the lowest wealth tertile (1st wealth tertile) was protective against seropositivity, risk ratio (RR) = 0.8 (95% CI = 0.7-0.9) (**Table 5**). Women who had history of tobacco use were found to be at lower risk of getting infection, RR = 0.8 (95% CI = 0.7-0.9). Furthermore, women using unimproved drinking water were at higher risk of infection, RR = 1.2 (95% CI = 1.1-1.4). In the follow-up survey, being in the 30-39 years age group, RR = 0.6 (95% CI = 0.4-0.9) and being underweight, RR = 0.4 (95% CI = 0.3-0.7) was found to be protective against the virus, while having more than 3 children in the household was associated with increased risk of infection, RR = 1.4 (95% CI = 0.9-2.0).

**Table 5:** Multivariable model showing risk factors associated with seropositivity among women at baseline and follow-up surveys

	Baseline		Follow-up	
**Factors**	**ARR (95% CI)**	**P-value**	**ARR (95% CI)**	**P-value**
				
**Number of living children in household, n (%)**				
≤2			Ref	
≥3			1.4 (0.9-2.0)	0.055
**Wealth quintile, n (%)**				
1st wealth tertile	0.8 (0.7-0.9)	0.018		
2nd wealth tertile	1.0 (0.9-1.2)	0.193		
3rd wealth tertile	Ref.			
Un-improved drinking water*	1.2 (1.1-1.4)	0.002		
**Women age (in years), n (%)**				
20-29			Ref	
30-39			0.6 (0.4-0.9)	0.029
≥40			0.7 (0.3-1.5)	0.443
Body mass index (kg/m^2^) (n = 1148)				
Underweight (BMI≤18.5)			0.4 (0.3-0.7)	0.001
Healthy weight (BMI>18.5 –<25)			Ref	
Overweight/obese (BMI≥25)			0.8 (0.6-1.2)	0.468
Tobacco user, n (%)	0.8 (0.7-0.9)	0.003		

ARR – absolute risk ratio, BMI – body mass index, CI – confidence interval

*Open well, closed well, tanker truck, small cart with tank, surface water (river/dam/lake/pond/stream/canal), bottled water, rainwater, other water source.

Similar to women, children who were in the lowest wealth tertile were found to be at a lower risk of infection, RR = 0.7 (95% CI = 0.5-1.0) at baseline. At the follow-up survey, children who did not have access to improved drinking water, RR = 1.4 (95% CI = 1.0-1.8) and whose fathers were uneducated were at greatest risk of being seropositive, RR = 1.3 (95% CI = 1.0-1.6) (**Table 6**). Being underweight, RR = 0.7 (95% CI = 0.5-0.8) and of age 24-36 months, RR = 0.7 (95% CI = 0.5-0.8) were found to be protective.

**Table 6:** Multivariable model showing risk factors associated with seropositivity among children at baseline and follow-up surveys

	Baseline		Follow-up	
**Factors**	**ARR (95% CI)**	**P-value**	**ARR (95% CI)**	**P-value**
**Age in months, n (%)**				
24-36			0.7 (0.5-0.9)	0.018
37-48			0.8 (0.7-0.9)	0.001
≥49			Ref.	
Underweight categories, n (%)*				
Normal (-1.99 to +6)			Ref.	
Underweight (-2.99 to -2)			0.7(0.5-0.8)	0.001
Severe underweight (≤-3)			0.8(0.6-1.1)	0.168
**Stunting categories, n (%)†**				
**Wealth tertiles, n (%)**				
1st wealth tertile	0.7 (0.5-1.0)	0.014		
2nd wealth tertile	0.8 (0.6-1.1)	0.713		
3rd wealth tertile	Ref.			
**Unimproved toilet facility‡**				
**Un-improved drinking water§**	1.4 (1.0-1.8)	0.059		
Household hunger*, n (%) (n = 902)				
**Highest grade or level of school completed by father, n (%)**				
No education	1.3 (1.0-1.6)	0.002		
Primary to secondary	Ref.			
**Father's occupation, n (%)**				
Government/private service/self employed	0.7 (0.6-0.9)	0.008	0.82 (0.70-1.0)	0.021
Daily wage earner/other (other work, does not work, farming, NA)	Ref.		Ref.	

ARR – absolute risk ratio, CI – confidence interval

*Data are available for age = 1097, weight = 1005.

†Data are available for height = 1010, age = 1097.

‡Dry toilet pump, bucket latrine, no toilet facility (uses open space or field, other toilet).

§Open well, closed well, tanker truck, small cart with tank, surface water (river/dam/lake/pond/stream/canal), bottled water, rainwater, other water source.

In both surveys, children whose fathers were employed (government, private or self-employed) were at a lower risk of infection, RR = 0.7 (95% CI = 0.6-0.9) and RR = 0.8 (95% CI = 0.7-1.0), respectively.

## DISCUSSION

We report a high seropositivity rate among women of reproductive age and children between March to June 2021 in a peri urban setting in Karachi, Pakistan. Almost half of the women and one-fifth of children in the AMANHI biorepository cohort showed evidence of past infection during the baseline survey that was conducted in the second quarter of 2021. We also report a much higher incidence rate of SARS-CoV-2 infection in subsequent serosurvey done during September to December 2021 in the same population. However, a greater proportion of those who tested positive seemed to have had asymptomatic infection.

In an unrelated serological survey done in November 2020 from the same study area, overall seroprevalence in all age groups, including men, was found to be 19.2% (95% CI = 16.0-22.9), similar to our baseline estimates. In the 15-49 age group in women, seropositivity was 22.5% (95% CI = 16.6-29.4) and in children under 5 years of age, it was 7.1% (95% CI = 2.3-15.8). Results from our surveys show a consistent increase over the previous estimates in line with the pandemic progress [17]. We found a substantial burden of COVID-19 infection among the young children in the follow-up survey. This is in line with the argument that. with increased rates of transmission, children may be equally vulnerable to the infection though they are more likely to develop asymptomatic or mild disease, as evidenced through our study. Women, too, had mostly asymptomatic infection. Global data also supported these findings. For example, Chatterjee et al. in India, found that the proportion of asymptomatic infection was 80% [18]. Similarly, a serial survey from Karachi reported that 7 out of 10 people who had the infection were not symptomatic [17]. Since the onset of the pandemic, COVID-19 has been known to cause a more severe disease among the elderly. Our cohort comprised young women with mean age of 29.3 ± 5.5 and children with mean age of 48.2 ± 15.1 in months. This may explain the greater proportion of asymptomatic cases in our participants.

In the follow-up serosurvey that was done four to nine months (median six months) after the end of the baseline, antibodies persisted for the majority of those who were initially found to be seropositive. This concurs with other studies that have shown a persistence of antibodies beyond 6-12 months after exposure to SARS-CoV-2 [19]. Although having COVID-19 does not necessarily translate into protection from subsequent infection, current evidence suggests that proportion of reinfections is still low [20]. Reinfections are mostly attributed to waning immunity or emergence of newer virus variants with mutations that allows for immune escape [21]. This makes the case for increasing the vaccination coverage in these communities.

We found that those having a low socioeconomic status had lower risk of SARS-CoV-2 infection at the baseline survey. This finding was consistent with a similar serological survey conducted in Bangladesh as part of the AMANHI study [22]. These results differed from global literature that shows higher rates of infection as well as infection severity among economically disadvantaged communities with overcrowding and poor sanitary conditions cited as possible reasons [23,24]. However, the low socioeconomic groups in our cohort are daily wage workers who work outdoors and were unemployed during the lockdown period. This could have led to low exposure and may explain the association with lower rate of infection at the baseline survey. In contrast, the follow-up survey was done well after the resumption of economic activities and similar rates of infection were seen across all socio-economic groups.

On the other hand, lack of access to improved drinking water was found to increase the risk of past infection with COVID-19 for both women and children at the follow-up survey. This may indicate overall low perception of hygiene and poor habits that could predispose to infection. Interestingly, during the same round, we found that when fathers were more educated, children were less likely to be seropositive. This can be explained by the role of fathers as household heads in possibly implementing preventive measures [25-27]. We also found that women who had a history of tobacco use in any form were less likely to be seropositive. Although some other studies have also showed smoking to be protective against SARS-CoV-2 infection, in our study most of the women were using tobacco in its smokeless form [28,29]. However, the exact underlying biological mechanism is unknown.

Our biggest strength was that we drew our sample from an established, well profiled prospective cohort, in which a weekly surveillance system made it possible to follow the participants for development of symptoms. We used testing kits with high degree of sensitivity and specificity and had a relatively large sample size for children under the age of 5 years. We had certain limitations. We did a qualitative assay for detection of anti-SARS-CoV-2 antibodies to assess the infection burden in our population, and hence, could not differentiate between current illness and past exposure. Moreover, we did not perform test for detection of neutralizing antibodies. However, we were able to collect population-based prospective data on seroprevalence and assess the seroconversion of a cohort of women and children, which could be used to plan context-specific strategies to containing COVID-19 pandemic in low and middle-income countries such as Pakistan.

## CONCLUSION

SARS-CoV-2 continues its rapid, and often, silent spread in the community, as evident through the high seroconversion rates shown in our study. This study highlights the value of community-based surveys in providing a more accurate estimate of the burden of disease in the community and in identifying community need, which in this case is, greater vaccination coverage. As more information about the long-term health effects of COVID-19 infection come to light, this information may be useful for policy makers and health planners in the future.

## Additional material:


Online Supplementary Document
