# Supplementary material for: Prevalence and risk factors of Severe Acute Respiratory Syndrome Coronavirus 2 infection in women and children in peri-urban communities in Pakistan: A prospective cohort study
Source: J Glob Health. 2022 Dec 17;12:05055. doi: 10.7189/jogh.12.95955 (PMC9757617; doi:10.7189/jogh.12.95955)
Supplement: Online Supplementary Document [file jogh-12-05055-s001.pdf]

Prevalence and risk factors of Severe Acute Respiratory Syndrome Coronavirus 2 infection in women and children in peri-urban communities in Pakistan: a prospective cohort study

Supplementary information

Authors

Nadia Ansari MSc[1*], M. Imran Nisar [1*], Farah Khalid MSc[1*], Usma Mehmood MSc[1], Asra Abeer Usmani MBBS[1], Fariha Shaheen MSc[1*], Aneeta Hotwani MPhil[1], Kehkashan Begum MPhil[1], Amina Barkatali MA[1], Sachiyo Yoshida PhD[2], Alexander A Manu[3], Sunil Sajawal PhD[4,5], Abdullah H Baqui PhD[6], Rajiv Bahl PhD[2], Fyezah Jehan MSc[1]**

Author Affiliations

1 Department of Pediatrics and Child Health, The Aga Khan University, Karachi

2 Department for Maternal, Child, Adolescents and Ageing Health, World Health Organization, Geneva, Switzerland

3 Department of Epidemiology and Disease Control, University of Ghana School of Public Health, Legon, Accra, Ghana

4 Center for Public Health Kinetics, Global Division, LGL Vinoba Puri, Lajpat Nagar II, New Delhi, India

5 Public Health Laboratory-IDC, Chake Chake, Pemba, Tanzania

6 Department of International Health, Johns Hopkins Bloomberg School of Public Health, Baltimore, Maryland, USA

*Joint first author

** Corresponding author

Table s1: Comparison of participants consented and refused for blood sample collection

	Consented	Refused	Total	P-value
	1414	485		
**Demographic Factors**				
**Number of household members**				
≤3	170 (12.0)	51 (10.5)	221 (11.6)	0.2
4-6	828 (58.6)	270 (55.7)	1098 (57.8)	
≥ 7	416 (27.2)	164 (33.8)	580 (30.5)	
**Wealth tertiles, n (%)**				
1st wealth tertile	466 (33.0)	167 (64.5)	633 (33.3)	0.6
2nd wealth tertile	468 (33.1)	165 (34.0)	633 (33.3)	
3rd wealth tertile	480 (34.0)	153 (31.6)	633 (33.3)	
**Type of toilet facility, n (%)**				
Improved toilet facility [1]	1183 (83.7)	402 (82.9)	1585 (83.5)	0.7
Unimproved toilet facility [2]	231 (16.3)	83 (17.1)	314 (16.5)	
**Drinking water, n (%)**				
Improved [3]	1121 (79.3)	397 (81.9)	1518 (79.9)	0.2
Un-improved [4]	293 (20.7)	88 (18.1)	381 (20.1)	
**Household Hunger*, n (%)**	n= 1472	n = 33	n = 1505	
No or mild hunger	1153 (78.3)	23 (69.7)	1176 (78.1)	0.2
Moderate to severe hunger	319 (21.7)	10 (30.3)	329 (21.9)	
**Total Coping Strategy Index (CSI) Score**	n= 1172	n= 327	n= 1499	
Mean ± SD	1.8 ± 4.8	1.8 ± 4.5	1.8 ± 4.8	0.1
**Woman related factors (Maternal and household characteristics)**				
**Woman Age categories, n (%)**				
20-29 years	505 (35.7)	252 (52.0)	757 (39.9)	<0.001
30-39 years	787 (55.7)	203 (41.9)	990 (52.1)	
≥ 40 years	122 (8.6)	30 (6.2)	152 (8.0)	
**Highest grade or level of school completed by woman n (%)**				
No education	735 (52.0)	257 (53.0)	992 (52.2)	0.4
Primary	334 (23.6)	106 (21.9)	440 (23.2)	
Secondary	272 (19.2)	88 (18.1)	360 (19.0)	
Post-secondary or higher	73 (5.2)	34 (7.0)	107 (5.6)	

**Woman occupation, n (%)**				
Housewife	1219 (86.2)	406 (83.7)	1625 (85.6)	0.2
Employed	195 (13.8)	79 (16.3)	274 (14.4)	
**Highest grade or level of school completed by husband n (%)**				
No education	711 (50.2)	228 (47.0)	939 (49.5)	0.5
Primary incomplete	297 (21.0)	109 (22.5)	406 (21.4)	
Completed primary	406 (28.7)	148 (30.5)	554 (29.2)	
**Husband occupation, n (%)**				
Govt/Private service	410 (29.0)	131 (27.0)	541 (28.5)	0.2
Daily wage earner	696 (49.2)	228 (47.0)	924 (48.7)	
Self-employed	238 (16.8)	92 (19.0)	330 (17.4)	
Other (Other work, does not work, farming)	70 (5.0)	34 (7.0)	104 (5.5)	
Total				
**Smoking, n (%)**				
No	1354 (95.8)	472 (97.3)	1826 (96.2)	0.1
Yes	60 (4.2)	13 (2.7)	73 (3.8)	
**Use of tobacco, n (%)**				
No	1024 (72.4)	337 (73.9)	1361 (72.8)	0.5
Yes	390 (27.6)	119 (26.1)	509 (27.2)	

[1] Flush or pour flush toilet, Pit latrine.

[2] Dry toilet pump, Bucket latrine, no toilet facility (uses open space or field, other toilet.

[3] Piped water into the dwelling, public tap, Tube well or borehole or hand pump.

[4] Open well, Closed well, Tanker truck, small cart with tank, Surface water(river/dam/lake/pond/stream/canal), Bottled water, Rainwater, Other water source.

Table S2 a: Distribution of common symptoms among women during baseline survey

Symptoms	n (%)
Headache	119(0.99)
Fever	111(0.93)
Body aches	77(0.64)
Cough	75(0.63)
Runny/stuffy nose	71(0.59)
Sneezing	40(0.33)
Fatigue	38(0.32)
Muscle aches	23(0.19)
Sore throat	22(0.18)
Dizziness or fainting	20(0.17)

Table S2b: Distribution of common symptoms among women during follow-up survey

Symptoms	n (%)
Body aches	412(1.08)
Headache	332(0.87)
Fever	303(0.8)
Fatigue	261(0.69)
Cough	139(0.37)
Runny/stuffy nose	135(0.36)
Dizziness or fainting	134(0.35)
Muscle aches	116(0.31)
Abdominal cramps or pain	77(0.2)
Sneezing	62(0.16)

Table S3 a: Distribution of common symptoms among children during baseline survey

Symptom	n (%)
Runny/stuffy nose	111(1.22)
Cough	87(0.96)
Fever	80(0.88)
Sneezing	35(0.38)
Diarrhoea	18(0.2)
Sore throat	13(0.14)
Nausea/ vomiting	12(0.13)
Headache	9(0.1)
Body aches	8(0.09)

Table S3b: Distribution of common symptoms among children during follow-up survey

Symptom	n (%)
Fever	208(0.72)
Cough	161(0.56)
Runny/stuffy nose	150(0.52)
Diarrhoea	57(0.2)
Skin rashes	48(0.17)
Sneezing	47(0.16)
Abdominal cramps or pain	31(0.11)
Body aches	30(0.1)
Headache	25(0.09)
